# Evaluating success rates of rotary NiTi versus reciprocating files in endodontic treatment

**DOI:** 10.6026/973206300220658

**Published:** 2026-02-28

**Authors:** Rutika Naik, Hiren Patel, Jayata Dhawan, Dipooja Patil, Monika Sharma, Priyanka Gupta, Miral Mehta

**Affiliations:** 1 Department of Conservative Dentistry and Endodontics, S B Patil Institute of Dental Sciences and Research, Bidar, India; 2 Department of Dentistry, Meharry School of Dentistry, Nashville, Tennessee, United States; 3 Department of Paediatric and Preventive Dentistry, MMCDSR, MULLANA, Ambala, Haryana, India; 4 Department of Conservative Dentistry and Endodontics, Bharati Vidyapeeth (Deemed to be University) Dental College and Hospital, Navi Mumbai, India; 5 Department of Dentistry, Government medical college, Bharatpur, India; 6 Department of Pediatric and Preventive dentistry, Swargiya Dadasaheb Kalmegh Smruti Dental College and Hospital, Nagpur, India; 7 Department of Pediatric and Preventive Dentistry, Karnavati School of Dentistry, Karnavati University, Gandhinagar, Gujarat, India

**Keywords:** Endodontic treatment, nickel-titanium files, rotary instrumentation, reciprocating motion, periapical healing, treatment outcome

## Abstract

Optimal file system selection remains critical in endodontic practice for effective root canal preparation in teeth with apical periodontitis. Therefore, it is of interest to compare rotary NiTi (ProTaper Gold) versus reciprocating (WaveOne Gold) systems in 224 teeth over 18 months. Both achieved comparable success rates (87.5% rotary vs. 89.3% reciprocating, p=0.672), with similar periapical healing and symptom resolution. Reciprocating files demonstrated shorter treatment times and fewer separations, improving procedural efficiency and safety. This advances endodontics by validating reciprocating systems as equally effective yet more efficient alternatives to rotary NiTi in primary treatments.

## Background:

Root canal treatment represents the cornerstone of managing pulpal and periapical pathology, with clinical success fundamentally dependent on effective chemo-mechanical preparation, disinfection and obturation of the root canal system [1]. The evolution of endodontic instrumentation has witnessed transformative advances from stainless steel hand files to sophisticated nickel-titanium (NiTi) systems that have substantially enhanced treatment predictability, efficiency and safety [2]. Contemporary endodontic practice offers clinicians diverse instrumentation options, yet optimal system selection remains subject to ongoing scientific debate and clinical preference. Nickel-titanium alloys revolutionized root canal instrumentation through their unique superelastic properties, enabling file designs capable of navigating curved canals while maintaining original canal anatomy [3]. The introduction of continuous rotary NiTi systems in the 1990s established new paradigms for root canal shaping, with sequential file systems providing controlled enlargement and improved debris removal compared to traditional step-back techniques [4]. These multi-file systems, including ProTaper Universal and subsequently ProTaper Gold, demonstrated enhanced cutting efficiency and flexibility while reducing procedural time. The subsequent development of reciprocating file systems represented another significant advancement, introducing single-file preparation concepts utilizing alternating clockwise and counter clockwise movements [5]. Reciprocating motion was proposed to reduce cyclic fatigue, extend instrument lifespan and decrease the risk of file separation by periodically releasing torsional stress accumulated during cutting [6]. WaveOne and subsequently WaveOne Gold exemplified this technology, offering simplified protocols with single-use instruments designed for complete canal preparation. The biomechanical principles underlying reciprocating motion differ fundamentally from continuous rotation. Reciprocating files engage dentin in cutting direction through greater clockwise movement and then disengage through smaller counterclockwise rotation, theoretically reducing instrument binding and screw-in effect [7].

Proponents argue this motion pattern enhances safety in curved canals while maintaining shaping efficiency comparable to rotary systems. Laboratory investigations comparing rotary and reciprocating systems have extensively evaluated parameters including shaping ability, centering ratio, transportation, debris extrusion and cyclic fatigue resistance [8]. These studies generally demonstrate comparable shaping outcomes between system types, with reciprocating files exhibiting superior fatigue resistance and rotary files potentially offering enhanced debris removal in certain configurations [9]. However, translation of laboratory findings to clinical outcomes requires validation through prospective clinical trials. Clinical success in endodontic treatment encompasses multiple dimensions including periapical healing, symptom resolution, functional tooth retention and absence of complications [10]. The periapical index (PAI) and cone-beam computed tomography periapical index (CBCTPAI) provide standardized frameworks for radiographic outcome assessment, enabling objective comparison across treatment modalities [11]. Long-term follow-up studies consistently demonstrate success rates of 85-95% for primary endodontic treatment, though outcomes vary based on preoperative diagnosis, tooth type and treatment variables. Contemporary systematic reviews and meta-analyses have attempted to synthesize comparative evidence between instrumentation systems [12]. However, most included studies focus on in vitro parameters or postoperative pain rather than long-term healing outcomes. The heterogeneity in study designs, outcome definitions and follow-up periods limits definitive conclusions regarding clinical superiority of either system type [13]. Several clinical studies have compared postoperative pain following rotary versus reciprocating instrumentation, with mixed findings ranging from equivalent outcomes to slight advantages for one system or another [14]. These investigations typically employ short follow-up periods insufficient for assessing periapical healing, which requires minimum 12-month observation for meaningful interpretation. The influence of instrumentation technique on microbial reduction and periapical tissue response remains incompletely characterized [15]. While laboratory studies demonstrate that both rotary and reciprocating systems effectively reduce bacterial loads when combined with appropriate irrigation protocols, the clinical significance of any differences requires investigation through outcome-based research. Procedural complications, particularly file separation, significantly impact treatment success and patient morbidity. The reported incidence of instrument fracture varies considerably across studies, ranging from 0.5% to 5% depending on file system, operator experience and clinical conditions [16]. Comparative data on separation rates between rotary and reciprocating files in clinical practice would inform instrument selection and risk management strategies. Economic and efficiency considerations additionally influence instrumentation selection in clinical practice. Single-file reciprocating systems potentially reduce instrument inventory requirements, simplify sterilization protocols through single- use paradigms and decrease procedural time [17]. However, these potential advantages must be balanced against clinical outcomes to establish true value propositions. Therefore, it is of interest to compare clinical and radiographic success rates between continuous rotary (ProTaper Gold) and reciprocating (WaveOne Gold) nickel-titanium file systems in primary endodontic treatment over 18-month follow-up, with secondary evaluation of procedural parameters, complications and patient- reported outcomes.

## Materials and Methods:

## Study design and ethical framework:

This prospective, parallel-group, randomized controlled clinical trial was conducted at the Department of Endodontics between February 2021 and November 2023.

## Sample size calculation:

Sample size determination was based on anticipated success rates of 88% for both treatment groups, with non-inferiority margin of 10% and equivalence hypothesis. Using alpha level of 0.05 and statistical power of 80%, minimum sample size of 98 patients per group was calculated. Accounting for potential 15% attrition over the 18-month follow-up period, target enrollment was established at 115 patients per group.

## Patient selection criteria:

Adult patients presenting with teeth requiring primary endodontic treatment due to pulpal necrosis with apical periodontitis were screened for eligibility. Inclusion criteria comprised: age between 18 and 65 years, presence of apical periodontitis confirmed by periapical radiolucency (periapical index score ≥3), pulp necrosis verified by negative response to cold and electric pulp testing, restorability of affected tooth following endodontic treatment, single-rooted or multi-rooted teeth with negotiable canals and willingness to attend scheduled follow-up appointments. Exclusion criteria included: teeth with previous endodontic treatment or surgical intervention, vertical root fractures, root resorption exceeding one-third of root length, periodontal probing depths exceeding 6 mm, systemic conditions affecting healing (uncontrolled diabetes, immunosuppression), pregnancy or lactation, chronic use of anti-inflammatory medications, teeth with calcified canals preventing instrument negotiation and open apex configuration.

## Randomization and allocation:

Eligible patients were randomly assigned to treatment groups using computer-generated randomization sequences stratified by tooth type (anterior/premolar/molar) in permuted blocks of four.

Allocation concealment was maintained through sequentially numbered opaque sealed envelopes opened immediately before treatment commencement.

[1] Group A (Rotary): Root canal preparation using ProTaper Gold rotary system (Dentsply Sirona, Ballaigues, Switzerland)

[2] Group B (Reciprocating): Root canal preparation using WaveOne Gold reciprocating system (Dentsply Sirona, Ballaigues, Switzerland)

## Clinical procedures:

All endodontic treatments were performed by three experienced endodontists who had completed minimum five years of clinical practice and demonstrated proficiency with both instrumentation systems. Operators were calibrated through treatment of 20 pilot cases prior to study commencement. Following local anesthesia administration, rubber dam isolation was established. Access cavity preparation was performed using high-speed diamond burs with water cooling. Canal orifices were located and initial patency confirmed using size 10 K-files. Working length determination employed electronic apex locator (Root ZX II, J. Morita, Kyoto, Japan) with radiographic confirmation. Glide path was established using Path File sequence (sizes 13, 16, 19) for rotary group and ProGlider (size 16) for reciprocating group.

## Rotary group protocol:

ProTaper Gold files were used in continuous rotation at 300 rpm and 2.5 Ncm torque following manufacturer recommendations. The sequence comprised SX (where indicated for coronal flaring), S1, S2, F1, F2 and F3 files as dictated by canal anatomy. Each file was used with brushing motion against canal walls, with irrigation between file changes.

## Reciprocating group protocol:

WaveOne Gold files were used in designated reciprocating motion using manufacturer-preset motor program. File selection (Small, Primary, Medium, or Large) was based on initial canal diameter assessment. Single-file preparation was completed with pecking motion advancing 3-4 mm before retraction and irrigation. Irrigation protocol was standardized across groups, comprising 5.25% sodium hypochlorite (3 mL between each file change), 17% EDTA for final rinse (1 minute) and saline final flush. Irrigation was delivered using side-vented 30-gauge needles positioned 2 mm from working length. Calcium hydroxide intracanal medication was placed for 14 days in all cases. At second appointment, obturation was performed using warm vertical compaction technique with gutta-percha and AH Plus sealer (Dentsply Sirona). Coronal seal was established with resin composite over 3 mm glass ionomer bases. Final restoration was completed within 30 days of obturation.

## Outcome assessment:

## Primary outcome:

Treatment success at 18 months, defined as absence of clinical signs and symptoms plus radiographic evidence of periapical healing (PAI score ≤2 or reduction by minimum 2 points from baseline).

## Secondary outcomes:

[1] Periapical index scores at 6, 12 and 18 months

[2] Clinical symptom resolution (pain, swelling, sinus tract)

[3] Procedural time (minutes)

[4] File separation incidence

[5] Postoperative pain (Visual Analog Scale 0-10) at 24, 48, 72 hours

[6] Analgesic consumption

[7] Patient satisfaction scores

Radiographic assessment utilized standardized periapical radiographs obtained using paralleling technique with customized positioning devices. Images were evaluated by two calibrated examiners blinded to treatment allocation, with disagreements resolved through consensus. Periapical index scoring followed established criteria (PAI 1-5).

## Statistical analysis:

Statistical analysis was performed using SPSS version 27.0 (IBM Corporation). Continuous variables were expressed as mean± standard deviation and compared using independent samples t-test or Mann-Whitney U test. Categorical variables were expressed as frequencies (percentages) and analyzed using Chi-square test or Fisher's exact test. Repeated measures ANOVA assessed changes over time. Logistic regression identified predictors of treatment success. Inter-examiner reliability was assessed using Cohen's kappa coefficient. Statistical significance was established at p<0.05.

## Results:

Of 246 patients screened, 230 met eligibility criteria and were randomized (115 per group). Six patients were excluded due to protocol violations or inability to establish glide path. Final analysis included 224 teeth in 218 patients (112 per group), with 6 patients contributing two teeth each. Follow-up completion rates were 96.4% at 18 months. Baseline demographic and clinical characteristics demonstrated adequate balance between groups (Table 1). Mean age was 38.4±11.6 years with slight female predominance (54.9%). Distribution across tooth types was comparable, with molars comprising 42.4% of treated teeth (Table 1). Treatment success rates at 18 months were comparable between groups, with 87.5% success in the rotary group and 89.3% in the reciprocating group (p=0.672) (Table 2). Complete periapical healing (PAI ≤2) was achieved in 79.5% and 81.3% of cases respectively. Partial healing with significant improvement was observed in an additional 8.0% and 8.0% of cases. Mean PAI scores decreased significantly in both groups from baseline to 18 months, with no significant intergroup differences in healing trajectory. Clinical symptom resolution was observed in 96.4% and 97.3% of cases in rotary and reciprocating groups respectively (Table 2). Procedural time was significantly shorter in the reciprocating group (Table 3). Mean instrumentation time per canal was 4.2±1.4 minutes for reciprocating versus 6.8±1.8 minutes for rotary preparation (p<0.001). Total treatment time including irrigation and obturation averaged 42.6±12.4 minutes for reciprocating versus 52.8±14.6 minutes for rotary procedures (p<0.001) File separation occurred in 4 cases (3.6%) in the rotary group compared to 1 case (0.9%) in the reciprocating group, though this difference did not reach statistical significance (p=0.176). All separated instruments were located in the apical third. Three of four separations in the rotary group occurred in severely curved molar canals. Postoperative pain scores were significantly lower in the reciprocating group at 24 hours (3.2±1.8 vs 3.8±2.1, p=0.024) and 48 hours (2.0±1.4 versus 2.4±1.6, p=0.048). Analgesic consumption was correspondingly reduced (3.8±1.8 vs 4.6±2.2 tablets, p=0.004). Flare-up incidence was comparable between groups. Patient satisfaction scores were significantly higher in the reciprocating group (8.7±0.8 vs 8.4±0.9, p=0.012), potentially reflecting shorter appointment duration (Table 3). Logistic regression analysis identified baseline PAI score (OR: 0.58, 95% CI: 0.38-0.88, p=0.012), lesion size >5 mm (OR: 0.42, 95% CI: 0.22-0.82, p=0.011) and presence of preoperative sinus tract (OR: 0.54, 95% CI: 0.28-0.96, p=0.038) as significant predictors of treatment outcome. Instrumentation system was not an independent predictor of success (OR: 1.18, 95% CI: 0.56-2.48, p=0.672).

## Discussion:

This prospective randomized controlled trial demonstrates equivalent clinical and radiographic success rates between continuous rotary and reciprocating nickel-titanium file systems in primary endodontic treatment of teeth with apical periodontitis. The observed success rates of 87.5% and 89.3% for rotary and reciprocating groups respectively align with outcomes reported in systematic reviews of contemporary endodontic treatment [1]. The equivalence in periapical healing outcomes confirms that both instrumentation kinematics achieve adequate chemo-mechanical preparation when implemented within standardized treatment protocols. The fundamental objectives of root canal preparation, including shaping efficiency, debris removal and access for irrigation, appear satisfactorily accomplished by both system types [2]. This finding has significant practical implications, enabling clinicians to select instrumentation based on preference, efficiency and economic considerations without compromising treatment outcomes. The healing trajectory observed in both groups followed expected patterns, with progressive improvement through 12 months and stabilization thereafter. Complete healing at 18 months (79.5% and 81.3%) corresponds with reported outcomes for teeth with preoperative periapical pathology [3]. The absence of significant intergroup differences at any follow-up interval reinforces the conclusion of therapeutic equivalence. Procedural efficiency significantly favoured reciprocating instrumentation, with 38% reduction in instrumentation time per canal. This advantage derives from the single-file preparation concept eliminating multiple file sequences and reducing instrument exchanges [4]. The simplified protocol additionally decreases cross-contamination risks and streamlines clinical workflow. In practice settings where appointment efficiency impacts productivity, this time savings represents meaningful value. The reduced postoperative pain observed with reciprocating instrumentation, though statistically significant, was clinically modest in magnitude (0.6 VAS points at 24 hours). This finding aligns with some previous investigations reporting mild advantages for reciprocating systems [5]. The mechanism may involve reduced debris extrusion, as reciprocating motion theoretically directs debris coronally during counter-clockwise rotation phases [6. File separation rates trended lower with reciprocating instrumentation (0.9% vs 3.6%), though statistical significance was not achieved, likely reflecting inadequate power for this secondary outcome. The biomechanical advantage of reciprocating motion in reducing cyclic fatigue accumulation has been extensively documented in laboratory studies [7]. Clinical validation of enhanced instrument safety represents an important consideration for risk management and treatment predictability. The comparable healing outcomes despite potential differences in shaping characteristics between systems merit consideration. Laboratory investigations have documented variations in canal transportation, centering ratio and residual dentin thickness between rotary and reciprocating files [8]. However, these geometric differences appear insufficient to influence biological outcomes when adequate disinfection is achieved through appropriate irrigation protocols. The standardized irrigation protocol employed in this study, featuring adequate sodium hypochlorite volume and EDTA application, likely represents a primary determinant of treatment success independent of instrumentation technique [9]. Contemporary understanding emphasizes irrigation as the critical component of root canal disinfection, with mechanical instrumentation primarily serving to create access and space for irrigant penetration. Logistic regression confirmed that preoperative disease severity, assessed through PAI score, lesion size and sinus tract presence, significantly influences treatment prognosis [10]. These findings align with established prognostic factors in endodontics and underscore the importance of preoperative assessment in outcome prediction. The absence of instrumentation system as an independent predictor further supports therapeutic equivalence. The single-use paradigm of reciprocating files offers advantages for infection control that deserve consideration beyond direct clinical outcomes [11]. Elimination of instrument reprocessing requirements reduces cross-contamination risks and simplifies sterilization protocols. Additionally, consistent performance characteristics through single-use may reduce variability associated with instrument fatigue accumulation. Patient satisfaction scores slightly favored reciprocating treatment, likely reflecting appreciation for reduced appointment duration [12]. Patient-centered outcomes increasingly influence treatment evaluation and procedural efficiency contributes to overall treatment experience. The high recommendation rates in both groups (94.6% and 97.3%) indicate favorable patient perceptions regardless of technique. The generalizability of findings requires consideration of study population characteristics. Inclusion of only teeth with apical periodontitis may limit applicability to vital pulp cases or retreatment scenarios [13]. Additionally, the involvement of experienced operators may not reflect outcomes achievable across all skill levels. Operator expertise represents a potential confounder in comparative instrumentation studies. All participating endodontists had extensive experience with both systems, minimizing learning curve effects [14]. However, operators transitioning to new systems may experience different outcome profiles during adaptation periods. The 18-month follow-up period, while adequate for meaningful outcome assessment, may not capture all treatment failures. Some investigations report continued healing through 24-48 months, particularly for larger lesions [15]. Extended follow-up would strengthen conclusions regarding long-term treatment durability. Cost-effectiveness analysis was beyond the scope of this investigation but warrants consideration in practice decisions. Reciprocating systems may offer cost advantages through reduced instrument inventory, elimination of multiple file purchases and efficiency gains [16]. Comprehensive economic evaluation incorporating direct costs, time savings and outcome equivalence would inform value-based decision-making. Emerging instrumentation technologies, including novel alloy formulations and heat-treated files, continue advancing the field [17]. Future comparative studies should evaluate these innovations against established systems to guide evidence-based practice evolution. Additionally, investigation of specific clinical scenarios, including severely curved canals, calcified canals and retreatment cases, would refine technique-specific recommendations [18].

## Conclusion:

Rotary (ProTaper Gold) and reciprocating (WaveOne Gold) nickel-titanium systems yielded equivalent clinical and radiographic success in treating apical periodontitis, with 18 month success rates of 87.5% and 89.3%, respectively. Reciprocating files demonstrated greater procedural efficiency, 38% shorter instrumentation time, slightly less postoperative pain and fewer file separations. Both systems are effective for primary endodontic treatment, with final outcomes more influenced by preoperative disease severity than by instrumentation kinematics.

## Figures and Tables

**Table 1:** Baseline demographic and clinical characteristics

**Paramete**r	**Rotary Group (n=112)**	**Reciprocating Group (n=112)**	**p-value**
Age (years), mean±SD	37.8±12.2	39.0±11.0	0.442
Gender (Male/Female)	48/64	53/59	0.486
Tooth type			0.824
- Anterior teeth	28 (25.0%)	26 (23.2%)	
- Premolars	36 (32.1%)	39 (34.8%)	
- Molars	48 (42.9%)	47 (42.0%)	
Jaw location (Maxilla/Mandible)	58/54	62/50	0.578
Mean baseline PAI score	3.86±0.72	3.78±0.68	0.398
Lesion size (mm), mean±SD	4.2±1.8	4.0±1.6	0.384
Presence of sinus tract	18 (16.1%)	16 (14.3%)	0.714
Preoperative pain (VAS)	4.6±2.4	4.4±2.2	0.518
Number of canals, mean±SD	2.1±1.0	2.0±0.9	0.432
PAI: Periapical Index;
VAS: Visual Analog Scale

**Table 2:** Treatment outcomes at follow-up intervals

**Outcome**	**Rotary Group (n=112)**	**Reciprocating Group (n=112)**	**p-value**
**6-Month Follow-up**			
Complete healing (PAI ≤2)	58 (51.8%)	62 (55.4%)	0.596
Improving (PAI reduction ≥2)	38 (33.9%)	34 (30.4%)	0.568
Unchanged	12 (10.7%)	14 (12.5%)	0.682
Worsening	4 (3.6%)	2 (1.8%)	0.412
**12-Month Follow-up**			
Complete healing (PAI ≤2)	76 (67.9%)	80 (71.4%)	0.562
Improving (PAI reduction ≥2)	18 (16.1%)	16 (14.3%)	0.714
Unchanged	10 (8.9%)	10 (8.9%)	1
Worsening	8 (7.1%)	6 (5.4%)	0.582
**18-Month Follow-up**			
Complete healing (PAI ≤2)	89 (79.5%)	91 (81.3%)	0.742
Partial healing (improved)	9 (8.0%)	9 (8.0%)	1
Unchanged/Failed	14 (12.5%)	12 (10.7%)	0.678
Overall Success Rate	98 (87.5%)	100 (89.3%)	0.672
Mean PAI at 18 months	1.68±0.84	1.62±0.78	0.582
PAI reduction from baseline	2.18±0.92	2.16±0.86	0.868
PAI: Periapical Index

**Table 3:** Procedural parameters and complications

**Parameter**	**Rotary Group (n=112)**	**Reciprocating Group (n=112)**	**p-value**
**Procedural Time**			
Instrumentation time/canal (min)	6.8±1.8	4.2±1.4	<0.001*
Total treatment time (min)	52.8±14.6	42.6±12.4	<0.001*
**Intraoperative Complications**			
File separation	4 (3.6%)	1 (0.9%)	0.176
Ledge formation	3 (2.7%)	2 (1.8%)	0.652
Perforation	1 (0.9%)	0 (0%)	0.316
**Postoperative Pain (VAS 0-10)**			
24 hours	3.8±2.1	3.2±1.8	0.024*
48 hours	2.4±1.6	2.0±1.4	0.048*
72 hours	1.2±1.0	1.0±0.8	0.098
7 days	0.4±0.6	0.3±0.5	0.186
Flare-up incidence	6 (5.4%)	4 (3.6%)	0.518
Analgesic tablets consumed	4.6±2.2	3.8±1.8	0.004*
Patient Satisfaction (1-10)	8.4±0.9	8.7±0.8	0.012*
Would recommend treatment (%)	94.60%	97.30%	0.318
*Statistically significant (p<0.05);
VAS: Visual Analog Scale
